# Germination and ultrastructural studies of seeds produced by a fast-growing, drought-resistant tree: implications for its domestication and seed storage

**DOI:** 10.1093/aobpla/plv016

**Published:** 2015-03-30

**Authors:** Helene Fotouo-M, Elsa S. du Toit, Petrus J. Robbertse

**Affiliations:** Department of Plant Production and Soil Science, University of Pretoria, Pretoria 0002, South Africa

**Keywords:** Deterioration, dormancy, lipid bodies, membrane leakage, protein bodies, seed, storage

## Abstract

Seed ageing during storage is one of the main causes of reduction in seed quality and this results in loss of vigour and failure to thrive. Finding appropriate storage conditions to ameliorate deterioration due to ageing is, therefore, essential. Ultrastructural changes in cellular organelles during storage and seed germination rates are valuable indices of damage that occurs during seed ageing. There is increasing interest in *Moringa oleifera* Lam. because of its multiple uses as an agroforestry crop. Seeds of this species lose their viability within 6–12 months of harvest but no scientific information is available on the longevity of seed stored in the fruit (capsules). In most undeveloped countries, seeds are still stored inside the fruit by traditional methods in special handmade structures. In this experiment we tried to simulate these traditional storage conditions. Capsules of *Moringa* were stored at ambient room temperature for 12, 24 and 36 months. The ultrastructure, solute leakage and viability of seed were investigated. The ultrastructure of 1-year-old seed showed no sign of deterioration. It was evident, however, that some cells of the 3-year-old seed had deteriorated. The remnants of the outer and inner two integuments that remain tightly attached to the cotyledons probably play a role in seed dormancy. No significant difference was found between germination percentage of fresh and 1-year-old seed. The germination percentage decreased significantly from 2 years of storage onward. The decrease in seed viability during storage was associated with a loss in membrane integrity which was evidenced by an increase in electrolyte leakage. Our findings indicate that the longevity of *M. oleifera* seeds can be maintained if they are stored within their capsules.

## Abstract (precis)

The inner layers of the seed coat that remain attached to the cotyledons probably play a role in seed dormancy of *Moringa oleifera*. Cotyledons of seeds stored for one year showed no sign of deterioration. In some cells of the three-year-old cotyledons, the membranes of the protein bodies were deteriorated. Cell deterioration was also marked by the collapse of the cell wall adjacent to the intercellular cavity. The decrease in seed viability during storage is associated with the loss in membrane integrity as confirmed by the increase in electrolyte leakage. The longevity of seeds can be extended if they are stored within their fruits under favourable conditions.

## Introduction

In seeds, age-induced deterioration results from various internal changes. Reserve substances may be altered so that they no longer supply the nutritional requirements of the embryo (Simola 1974). Membrane aberrations are said to increase with seed ageing (Berjak and Villiers 1970) and result in increased leakage of metabolites and ions (Roberts and Ellis 1982; Ouyang *et al*. 2002). As seed deterioration increases, the rate of germination decreases, and production of weak seedlings with loss of vigour increases progressively. According to Garcia de Castro and Martinez-Honduvilla (1984), seed ageing is a complex process, so it is essential to investigate this process at the subcellular level in order to understand the best conditions for seed storage. Despite the publication of numerous papers on seed ultrastructure at various phases of development during the last two decades (Berjak *et al.* 1994; Algan and Bakar 1997; Wang and Berjak 2000; Wiśniewska *et al.* 2006; Teixeira and Machado 2008; Egorova *et al.* 2010; Moura *et al*. 2010; Chan and Belmonte 2013; Hu *et al.* 2013; and citations therein) knowledge of the ultrastructural changes that occur during seed storage is still insufficient. The complexity of seed tissues and the difficulties in preparing samples for microscopy have until now precluded ultrastructural studies on *Moringa oleifera* Lam. seeds.

*Moringa oleifera*, or miracle tree, is known in many parts of the world for its multiple uses as an agroforestry crop. The leaves, flowers and immature fruits are edible (Anwar and Bhanger 2003). The leaves are a good source of protein, vitamin A, B and C and minerals such as calcium and iron (Dahot 1988). A number of medicinal properties have been ascribed to various parts of this tree (Kumar *et al*. 2010). *Moringa* seeds contain ∼35–40 % oil, commercially known as ‘Ben oil’ (Anwar and Rashid 2007). *Moringa* domestication and commercialization is still a challenge as its agronomic properties have not been well elucidated. It is a perennial tree that in most cases is grown as an intercrop or in an agroforestry set-up and only bears fruit (capsule) once a year. The crop is mainly propagated by seed (Radovich 2012). Many studies (Bezerra *et al*. 2004; Madinur 2007; De Oliveira *et al*. 2009) have found that *M. oleifera* seeds lose their viability and vigour within 6–12 months depending on the conditions in which they are stored. High-quality seed is essential for most crops including agroforestry crops like *Moringa*. Seed ageing is one of the main causes of reduction in seed quality (FAO/IPGRI 1994). Finding appropriate storage conditions to ameliorate deterioration is essential.

Simple techniques such a hessian sacks, cotton bags, paper containers, cardboard, aluminium cans and foil, glass jars and plastic film stored at ambient temperature have been used to maintain seed viability in both domesticated and wild sources since early times (Willan 1985; Ellis *et al.* 1996; Akai 2009). In sub-Saharan Africa and other developing countries around the world, traditional methods of seed storage have been used for many years with little or no modification (Mannan and Tarannum 2011). Traditional storage facilities such as underground pits and different types of solid wall bins (made of timber, earth and stone) are usually used by subsistence or small-scale farmers to keep part of their products to be used as seed in the following planting season (Ali Ahmed and Ahmed Alama 2010). Farmers must also be able to store their products until the next successful harvest and this might be more than a year in the case of crop failure (Blum and Bekele 2000).

Seed stored within the pods of some legumes can withstand infestation by storage pests and tend to extend seed viability. This relatively positive feature is often used by farmers to minimize crop damage by storing in pods. The method can be applied to a variety of edible legumes, and is particularly valid for bambara nuts, groundnuts and cow peas (Odogola 1994). Cow pea pods are usually hand-picked when mature, bagged and then hauled to a place where they are stored for a variable period until they are threshed. After threshing, the seed becomes more exposed to post-harvest insect pests and is vulnerable to these insects throughout subsequent storage (Murdock *et al.* 2003). It is with this background in mind that this study was initiated.

No study has investigated the effects of traditional methods of storage, which are used primarily by most small and poor farmers. The aim of this study was to determine the germination percentage of *M. oleifera* seed that was left within the fruit (capsule) and stored at ambient room temperature and to provide details on ultrastructural changes that occur during seed ageing.

## Methods

### Seed material

Fruits (capsules) of *M. oleifera* were harvested during 2009 and 2011 from an orchard growing on the Experimental Farm of the University of Pretoria (25°45S, 28°16E). They were bagged in open poly mesh bags and stored at ambient room temperature (annual average temperature: 23–25 °C) for 12, 24 and 36 months. Seeds from the 2011 harvest were assessed after 12 and 24 months and seeds from the 2009 harvest were assessed after 36 months. After threshing, only seeds with the physical characteristics of maturity were selected for the study.

### Seed morphology and ultrastructural studies

The outer part of the seed coat of fresh, 12-month-old seeds and 36-month-old seeds was removed. Seeds were imbibed for 24 h after which the samples were divided into two sub-samples: one for light microscopic and another for transmission electron microscopic examination.

#### Seed morphology

Morphological characteristics of the seed were observed using a dissecting microscope. The cotyledons and the embryo were carefully separated. Photographs were taken with the Zeiss Discovery V20 stereo microscope (Jena, Germany).

#### Light microscopy

The cotyledons of the seeds were separated and fixed with FAA (formaldehyde, acetic acid and ethanol) inside polytops. Thereafter, samples were dehydrated in an ethanol series (30, 50, 70 and 100 %) and each concentration was replaced after 24 h, except for 100 % alcohol that was repeated. Ethanol was extracted from the samples with a series of xylene (30, 50, 70 and 100 %). After gradual wax infiltration, the samples were embedded in pure wax and mounted on stubs. A microtome (2040 Autocut Sterea Star Zoom, Reichert Jung-0.7× to 42 × 570, Leica, Vienna, Austria) was used to cut samples at ∼10 μm. Sections were stained with saffranin, counterstained with fast green and mounted in DPX mountant. Pictures were taken with a digital camera (Nikon DXM 1200) mounted on a Zeiss Discovery V20 stereo microscope and light microscope (Nikon/SMZ-1, Japan).

#### Transmission electron microscopy (TEM)

Small samples (2 × 2 mm) were excised from the cotyledons to include the epidermis and sub-epidermal layers as well as samples from the central part of the cotyledons and then prepared for TEM, according to Coetzee and Van Der Merwe (1996). The samples were fixed for 3 days in 2.5 % glutaraldehyde in 0.075 M phosphate buffer (pH 7.4), after which they were rinsed three times (10 min each) in 0.075 M phosphate buffer. Samples were further post-fixed in 0.5 % aqueous osmium tetroxide for 2 h and thereafter rinsed three times with distilled water. This was followed by dehydration in an ethanol series (30, 50, 70, 90 and 100 %), infiltrated with 30 and 60 % quetol for 1 h each and pure quetol for 4 h and then polymerized at 60 °C for 39 h. Ultrathin sections were prepared using a Reichert Ultracut E ultramicrotome (Vienna, Austria). The sections were stained with 4 % aqueous uranyl acetate and lead citrate (Reynolds 1963) for viewing and photographing with a JEOL JEM-2100F transmission electron microscope (JEOL, Tokyo, Japan).

### Seed germination

Seeds of all the treatments were germinated using the same procedure. Each treatment consisted of four replicates of 50 seeds. Prior to germination, the outer part of the seed coat was removed to minimize fungal attack. The inner part of the seed coat containing remnants of the inner integument remained tightly attached to the cotyledons and could not be removed without damaging the cotyledons. The seeds were then disinfected in 1 % of sodium hypochlorite for 25 min and rinsed three times in sterile distilled water in a the laminar airflow hood. Seeds were germinated according to ISTA (2006) procedure. The 50 seeds of each replicate were distributed on paper towel rolls soaked with 70 mL of water and incubated in controlled temperature chambers at alternative temperatures of 20/30 °C. Germinated seeds were counted for the first time after 7 days and the last count after 14 days.

#### Tetrazolium test (TTA)

The TTA was conducted on seeds that did not germinate to test for possible lack of viability. The test was done according to ISTA (ISTA 2006). Tissues necessary for development of seed into seedling should stain red.

### Electrolyte leakage

Solute leakage of seeds was estimated by placing one seed gram (i.e. without seed coat) into 10 mL of distilled water for 24 h at 25 °C (Rao *et al.* 2006). This was replicated at least four times (≥20 seeds/treatments). Electrical conductivity of the water containing the leakage was measured with a conductivity meter (Mettler Toledo, 8603. Switzerland).

### Statistical analysis

Germination percentage and electrical conductivity were statistically analysed using STATISCA software (STATISCA 12, Statsoft 2013). A one-way analysis of variance (ANOVA) was performed to determine the statistical difference between germination percentage and electrolyte leakage as influenced by storage duration. The Duncan post-hoc test (*P* < 0.05) was used to check the significance between groups.

## Results

### Seed morphology and ultrastructure

#### Seed morphology

**Figure 1.**
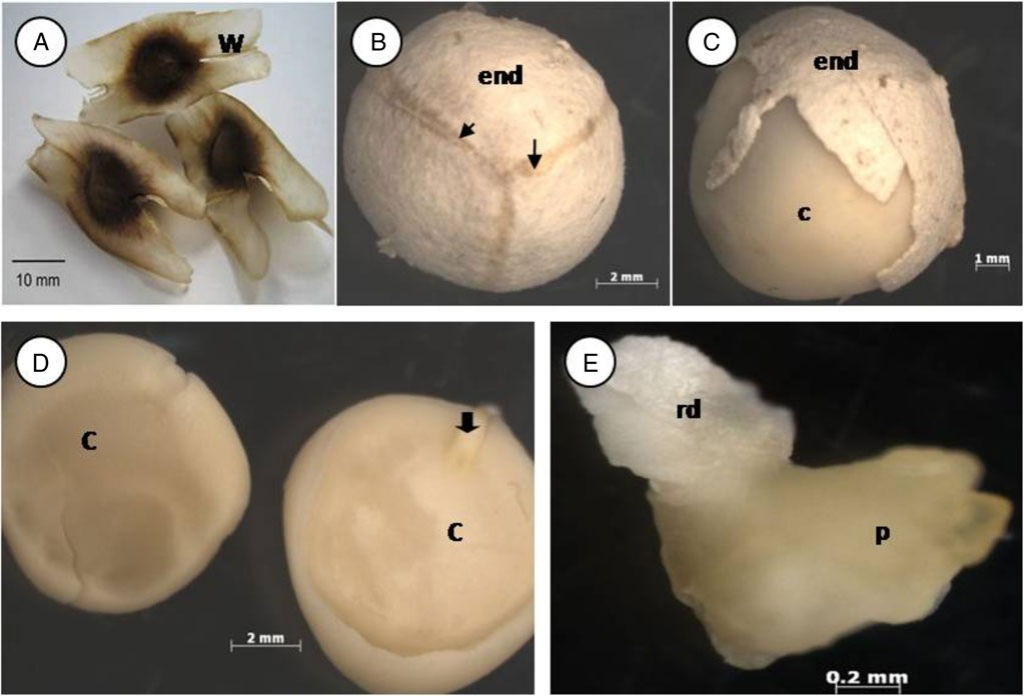
*Moringa* seed morphology. (A) Seed with seed coat and wings. (B) Seed with internal seed coat and vascular bundle. (C) Seed with partially removed internal seed coat. (D) Separated cotyledon. (E) Embryo axis. Wing (w), vascular bundle (arrowhead), cotyledon (c), endotesta (end), embryonic axis (black arrow), radical (rd), plumule (p).

The general morphology of *M. oleifera* seed is represented in Fig. 1. The seeds are round, protected with a brownish seed coat containing three white wings (Fig. 1A). When trying to remove the seed coat, the inner part containing remnants of the outer integument and the inner integument remains tightly attached to the cotyledons as a creamy white layer with three stripes containing vascular bundles below the wings (Fig. 1B and C). The diminutive embryonic axis (1.5–2 mm) is located in the small cavity between the two cotyledons near the micropilar region (Fig. 1D). The embryonic axis has a distinguished radicle (whitish) and plumule that is slightly split into two ends, representing the primordial of the first leaves (Fig. 1E).

#### Light microscopy

**Figure 2.**
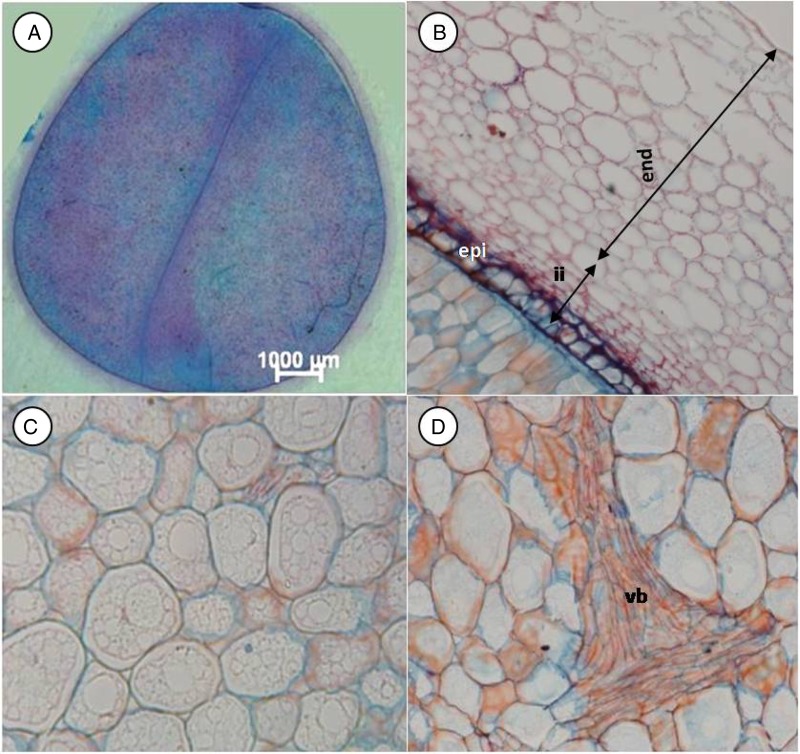
Light micrographs of parts of *M. oleifera* seed. (A) Transverse section of cotyledons and remnant of seed coat. (B) Endotesta, inner integument (ii) covering the outer cell layer of the cotyledon. (C) Sub-epidermal cell layers of cotyledon. (D) Central tissue of cotyledon. Endotesta (end), epidermis of inner integument (epi), vascular bundle (vb).

The transverse section of a mature *M. oleifera* seed reveals cotyledons enclosed by inner endotesta and inner integument composed of different layers of non-living cells (Fig. 2A and B). As described by Muhl (2014), the endotesta originates from parts of the outer integument and is made up of layers of elongated, thickly reticulate cells. Below the endotesta is the compressed inner integument with prominent epidermis cells having a thick cell wall. Cells of the cotyledons are isometric or roundish in shape and filled with storage material. Vascular bundles are absent in sub-epidermal tissue (Fig. 2C) and present in the central layers (Fig. 2D). No difference was visible between seed of different ages with the light microscope.

### Transmission electron microscope (TEM)

#### Cotyledons

**Figure 3.**
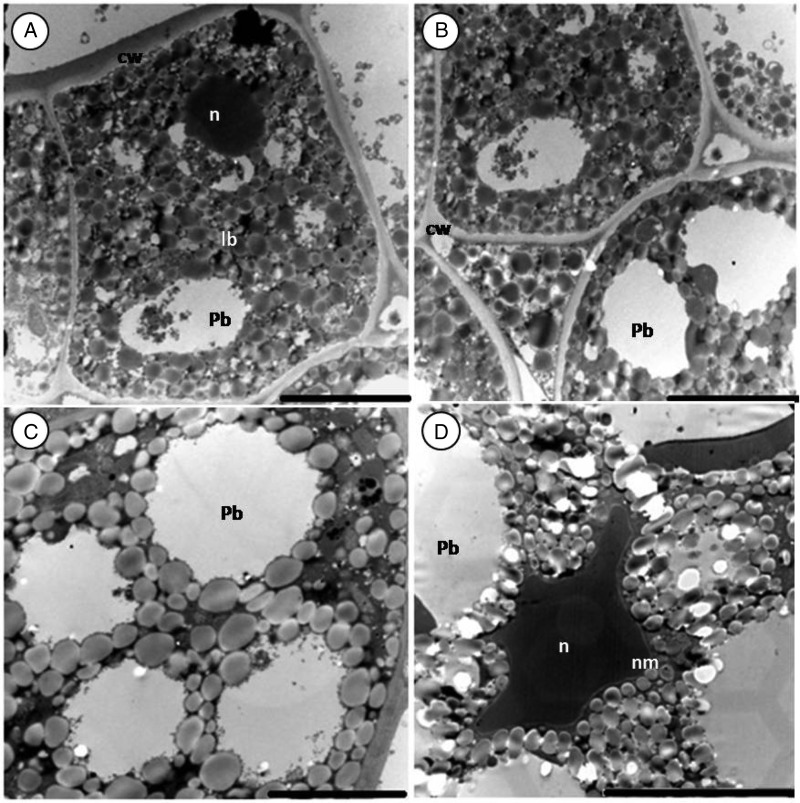
Sections of a fresh (control) *M. oleifera* seed (cotyledons). (A) Epidermis with fewer protein bodies and a thick external cell wall. (B) Epidermal and sub-epidermal cell. (C and D) Sub-epidermal cells filled with large protein and lipid bodies. Note the electron-dense nucleus (n in D) of irregular shape with well-defined membrane squeezed between storage materials. Lipid bodies (lb), protein bodies (pb), nuclear membrane (nm). Scale bars: A–C (5 µm); D (10 µm).

**Figure 4.**
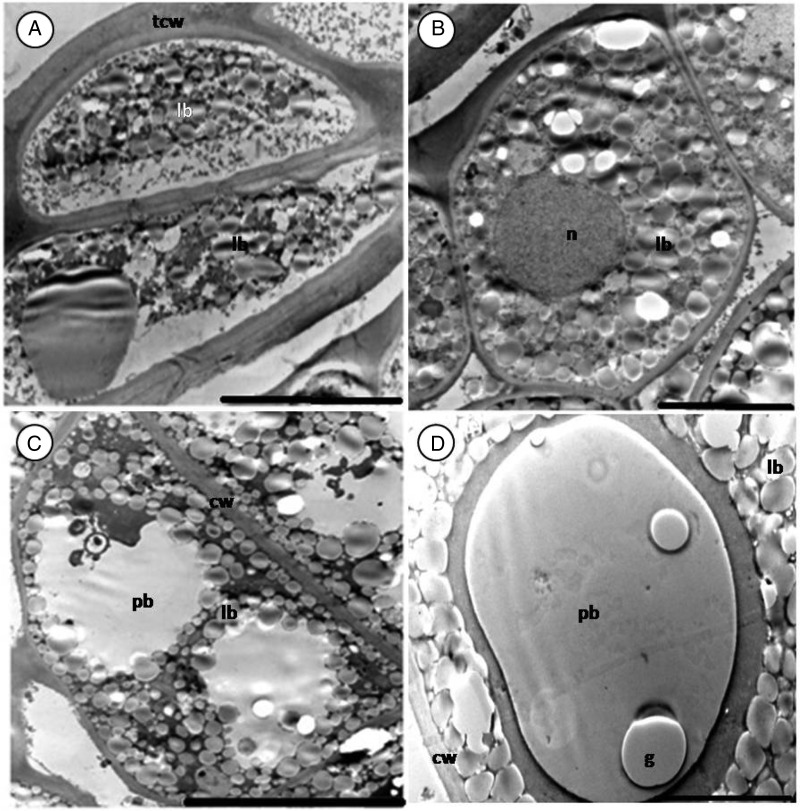
Section of 1-year-old seed (cotyledon) of *M. oleifera*. (A) Epidermis cells of the inner integument. Note the thick cell wall. (B) Epidermal cells of cotyledons. (C) Sub-epidermal cell filled with protein and lipid bodies. (D) Protein bodies embedded in the cytoplasm. Note globoid (g) inclusions in protein bodies. Scale bars: A (10 µm); B–D (5 µm).

As seen with the light microscope, the epidermal cells of the inner integument have thick cell walls (Fig. 4A). A small nucleus is present at the centre of some cells (picture not shown). Below the inner integument are the epidermal cells of the cotyledon (Figs 3A, 4B and 7A). They are occupied by lipid bodies and a conspicuous nucleus. Protein bodies are seldom present and plastids are absent. No difference was found between epidermal cells of seeds from different storage periods.

**Figure 5.**
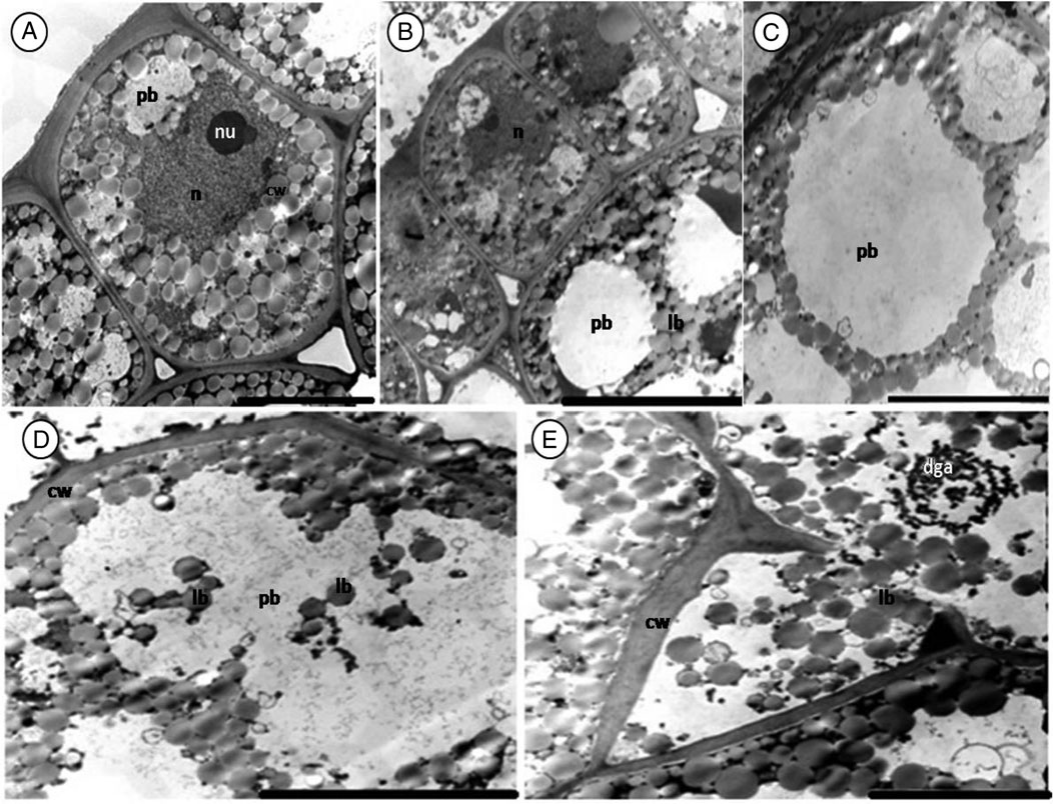
Sections of 3-year-old seed (cotyleton) of *M. oleifera*. (A) Epidermal cells with few protein bodies between lipid bodies. (B) Epidermal and sub-epidermal cells. (C) Sub-epidermal cells. Note the presence of protein body membrane. (D and E) Deteriorated sub-epidermal cells. (D) Membrane has deteriorated and protein appears coalescent. (E) Broken cell wall allowing merging between the plasmalemma of intercellular space and adjacent cells. Note the presence of a deteriorated Golgi apparatus (dga). Scale bars: A and E (5 µm); B–D (10 µm).

The structure of sub-epidermal cells of the cotyledon was uniform. Lipid bodies surrounded the protein bodies and filled most of the remaining space in all healthy cells, irrespective of the age (Figs 3C, 4C–D and 5C–D). The lipid bodies are numerous, round to oval in shape, small in size and have a uniform grey interior. Protein bodies occupy most of the cytoplasm volume. Globoid inclusions were present occasionally (Fig. 4D). Most organelles were not noticeable in the cotyledon as the entire cytoplasm was filled with storage materials, except for the irregularly shaped nucleus with a well-defined nuclear membrane (Fig. 3D).

Sub-epidermal cells of the 1-year cotyledon showed no sign of deterioration (Fig. 4). In the 3-year-old cotyledon, the deterioration was not uniform. Some cells seemed to be still perfectly healthy (Fig. 5C) while damage was noticeable in others (Fig. 5D and E). The membrane of the protein body was deteriorated, causing lipid bodies to enter the protein body (Fig. 5D). Cell deterioration was also marked by the collapse of the cell wall adjacent to the intercellular cavity (Fig. 5E). A degraded Golgi apparatus next to the broken cell wall (Fig. 5E) was observed.

**Figure 6.**
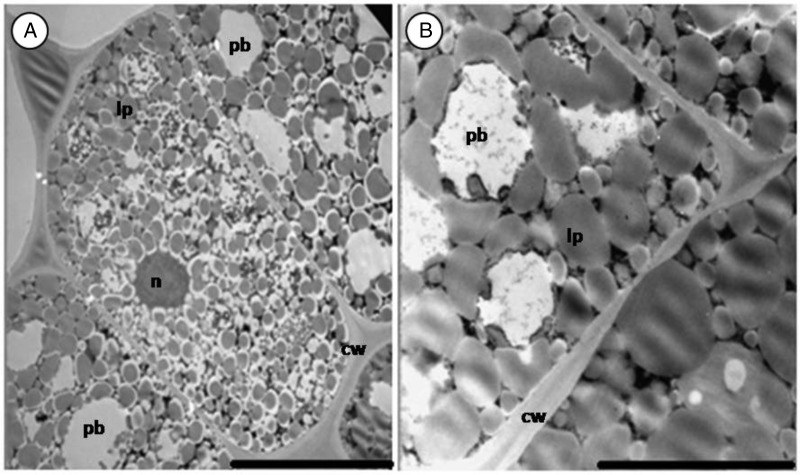
Section of central cotyledon cells of 3-year-old *M. oleifera*. (A) Scattered lipid bodies occupy most of the cell volume. (B) Protein bodies are small and disrupted. Scale bars: A and B (10 µm).

In the 3-year-old seeds, the central cells of the cotyledons were almost completely filled with scattered oil bodies (Fig. 6A and B). The protein bodies were disrupted and smaller in size compared with those found in sub-epidermal cells. No sign of deterioration was evident in central cells of cotyledons of the 1- and 3-year-old seeds.

#### Embryo axis

**Figure 7.**
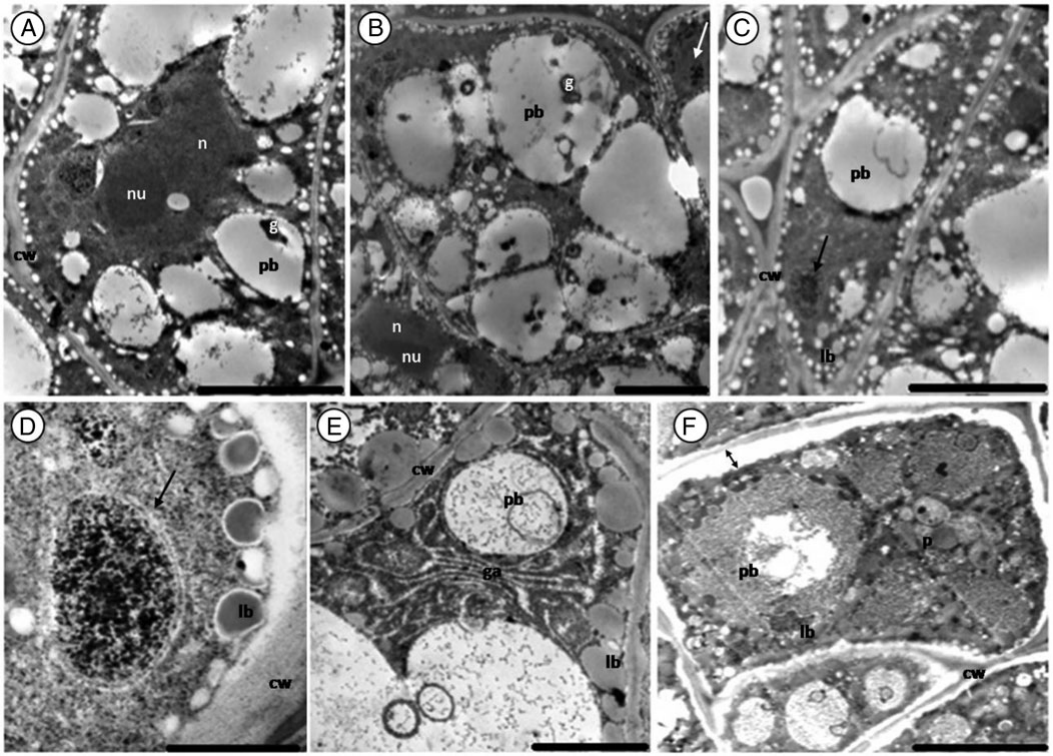
Section of embryo axis of *M. oleifera*. (A–C) Healthy cells of fresh, 1- and 3-year-old seeds. (D) Mitochondria with double membrane (arrow). (E) Golgi apparatus (ga) occupying part of the cytoplasm. (F) Deteriorated cells of embryo axis of 3-year-old seed. Note the withdrawal of the plasmalemma (double arrow). Scale bars: A–C and F (5 µm); D (1 µm); E (2 µm).

The main storage material in the embryo axis was protein bodies. They occupied more of the cell volume compared with the sub-epidermal cells of the cotyledons (Fig. 7A–C). Globoid inclusions were small and numerous when present. Lipid bodies formed a wall lining along the plasma membrane and built a single layer around protein bodies. The cells of the embryo axis were not totally filled with storage material as was the case with cotyledon cells; the remaining space was filled with ground substances or cytoplasm and various organelles. A prominent nucleus with well-defined nucleolus was present in most cells. Mitochondria with cristae and a double membrane (Fig. 7D) were present in cells of all ages. Golgi apparatus was spotted in cells of fresh embryo (Fig. 7E).

As was the case with sub-epidermal cells of the cotyledon, some cells of the embryo axis still maintained the ultrastructure of healthy cells while others showed some anomalies (Fig. 7F). In some cells, the cytoplasm had shrunk and detached from the cell wall. The extent of the detachment varied from one cell to another; some were moderate and others severe.

### Seed viability and electrical conductivity

The mean germination percentage and the electrolyte conductivity of seed stored for different periods are represented in Table 1. The germination percentage was determined to be 67 % for the fresh seeds and decreased to 16 %, while the seed viability (germination percentage + TTA test) was initially 80 % and declined to 18 % after the fruits were stored at ambient temperature for 3 years. Seeds maintained a high germination percentage until the end of 1 year of storage; after 2 years seed viability declined significantly (*P* < 0.05). The electrical conductivity of seed increased significantly as the storage duration increased.

**Table 1.** Average germination percentage, with or without tetrazolium test (TTA) and electrical conductivity of seed, left in capsules (fruit) for different storage periods. Data shown are means of four replicates ±SE. Averages with different letters are significantly different (*P* ≤ 0.05).

Storage duration (years)	Germination		Electrical conductivity (µS/cm)
	Germ %	Germ % +TTA	
Fresh (0)	67 ± 1.4^a^	80 ± 0.96^a^	21.83 ± 0.7^a^
1	64 ± 1.7^a^	72 ± 1.31^a^	32.945 ± 0.5^b^
2	40 ± 3.1^b^	46 ± 3.6^b^	88.8 ± 3.01^c^
3	16 ± 0.85^c^	18 ± 0.62^c^	101.88 ± 1.76^c^

## Discussion

### Morphology and ultrastructural studies

#### Cotyledons

The ultrastructure of cotyledonary tissues of *M. oleifera* is similar to those of other oily seeds (Muller *et al*. 1975; Young *et al*. 2004; Kuang *et al.* 2006; Donadon *et al*. 2013) and other seeds in general (Cecchifiordi *et al.* 2001; Pinzón-Torres *et al*. 2009). The seeds are filled with storage material that hampers the visualization of organelles, except for the lobed nucleus and plastids that were spotted in some cells. The scarcity of organelles may suggest low metabolic activity and the restricted function of cotyledons as storage organs (Pinzón-Torres *et al*. 2009). The organization of storage material in different tissues is probably determined by the role they play and the order in which they are used during germination. Starch grains are totally absent in the cotyledons.

The ultrastructure of the 1-year-old cotyledons was similar to that of the fresh cotyledons. In the sub-epidermal cells of the 3-year-old seed, the membrane of the protein bodies had ruptured, leading to the coalescence of protein bodies into a large confluent masse and the engulfment of lipid bodies by the protein substances. Similar observations have been reported by Smith (1978) and Dawidowicz-Grzegorzewska and Podstolski (1992), in artificially aged lettuce and *Brassica napus* seeds. Protein bodies contain several hydrolytic enzymes, among them phosphatases. The release of their contents into the cytosol as a result of membrane deterioration can cause localized cellular autolysis (Dawidowicz-Grzegorzewska and Podstolski 1992). The breakdown of the cell wall and the disintegration of the Golgi apparatus were also evident in severely deteriorated cells. Breakages of cell wall have not been reported in previous studies. One would be tempted to assign this breakage to sample preparation but with the presence of disintegrated Golgi apparatus next to the broken cell wall, the breakage was likely caused by deterioration during ageing. Cellular membrane damage is mediated by oxidative attack, which promotes phospholipid degradation and the loss of membrane organization (Pukacka and Ratajczak 2007). The decrease in size of lipid bodies and their coalescence have been reported in aged and non-viable seed by other authors (Garcia de Castro and Martinez-Honduvilla 1984; Neya *et al.* 2004; Walters *et al*. 2005; Donadon *et al*. 2013). Lipid bodies of *M. oleifera* remained intact after 3 years of storage.

#### Embryo axis

As found for *M. oleifera*, lipid and protein bodies in cells of the embryo axis have been reported in the seed of many species such as *B. napus* (Kuraś 1984; Dawidowicz-Grzegorzewska and Podstolski 1992), *Origananum majorana* (Wiśniewska *et al*. 2006), *Amaranthus hypocondriacus* (Ciombra and Salema 1994), *Picea mariana* (Wang and Berjak 2000) and carrot (Dawidowicz-Grzegorzewska 1997). Germination is initiated in the embryo. Protein content in the embryo is higher than that in the cotyledon. The protein reserve function is to supply amino acids for the formation of enzymes during germination, which are used by the cell for hydrolysis of storage materials (Pernollet 1978). Lipid bodies in embryo axis cells of a number of species are thought to serve as reservoirs during germination (Pinzón-Torres *et al*. 2009).

Damage observed in the embryo was different from that of the cotyledons. The separation of the plasmalemma from the cell wall in non-viable seeds has been observed in many species (Anderson 1970; Garcia de Castro and Martinez-Honduvilla 1984) but has also been linked to imbibition damage (Hoekstra *et al*. 1999). Thus membrane withdrawal can occur either as a result of ageing or during sample preparation (fixation). In this study, the storage may have played a huge role as the leaching of cellular constituents in the 3-year-old seeds was found to be significantly higher than in other seeds. The nuclei maintained their morphology in the old seed even when some parts of the cells were damaged. The resistance of nucleus to deterioration during senescence has also been reported by Simola (1974).

### Seed viability and electrical conductivity

Nouman *et al.* (2012) observed that dehulling of *Moringa* seed did not significantly increase the germination rate while Mubvuma *et al.* (2013) reported that they scarify the seed before planting in order to increase water uptake. According to our observations (Figs 3B and 5A–B), it is not possible to remove the entire seed coat without damaging the seed, which suggests that for any germination test, research should be more specific about the removal of the seed coat.

Initial germination percentages of 84 and 93 % for *M. oleifera* have been reported by Bezerra *et al.* (2004) and Madinur (2007), respectively. The germination percentages obtained in the present study compare well with dehulled seeds of Nouman *et al.* (2012). According to the TTA test 13 % of the seeds were dormant, probably as a result of the remnants of the seed coat remaining attached to the cotyledons. This finding is supported by Nouman *et al.* (2012), who reported that priming the seed with *Moringa* leaf extract improved the emergence of *Moringa* seedlings. Similar observations were made by Mubvuma *et al.* (2013); they found an increase in germination percentages after storing seeds for 60 days at 25 and 35 °C, but they argue that *Moringa* seeds are non-dormant because fresh seeds can readily germinate after exposure to favourable conditions and that the improvement after exposure to high temperature may be explained by genetic adaptation of seed within their centres of origin where the temperature ranges between 30 and 35 °C. By the same logic it is likely that *Moringa* seeds acquire dormancy when they are grown in an environment with an average annual temperature below 30 °C. High temperatures and priming treatment are some of the methods used to break seed dormancy.

De Oliveira *et al.* (2009) reported that *M. oleifera* seeds retain their viability after 6 months of storage at ambient temperature, irrespective of the type of storage. This was supported by a previous study done by Bezerra *et al.* (2004). The authors also observed a 65 % decrease in germination percentage after 12 months and a complete loss of viability after 24 months. Madinur (2007) reported a continuous significant decrease from 2- to 12-month storage at room temperature. In the present study, the seed viability remained almost unchanged after 12 months and maintained 46 % viability after 24 months of storage. These values are considerably higher than those found in studies where seeds were separated from the capsules before storage. Seed viability decreases as a result of loss of membrane integrity (Fig. 6D), which is supported by the increase of solute leakage (Table 1). Higher leachates have also been recorded in other stored high oil content seed such as *Carthamus tinctorius* (Alivand *et al.* 2012) and sunflower (Kallapa 1982). According to these authors the higher electrolyte leakage in oily seed is a result of the leaching out of free fatty acid during storage.

## Conclusions

The remnants of the outer integument and the inner integument that remain tightly attached to the cotyledons when trying to remove the seed coat probably play a role in seed dormancy. The decrease in seed viability during storage is associated with the loss in membrane integrity and it is confirmed by the increase in electrolyte leakage. The longevity of *M. oleifera* seeds can be extended if they are stored within their capsules (fruit). Capsule storage does not require additional equipment and can be adopted by small farmers. *Moringa oleifera* seed are high in oil content, making fixative infiltration very difficult and therefore affecting the quality of the micrographs.

## Sources of Funding

This work was funded by the National Research Foundation, South Africa.

## Contributions by the Authors

All authors contributed extensively to the work presented in this paper. H.F.-M. designed, performed experiments, analysed data and wrote the manuscript. E.S.d.T. and P.J.R. were involved in designing and supervising data analysis, and edited the manuscript.

## Conflict of Interest Statement

None declared.
